# From the 20th of September to the 20th of October 1799; Being the Result of the Public and Private Practice of a Physician at the West End of the Town

**Published:** 1799-11

**Authors:** 


### STATE OF DISEASES IN LONDON,

*From the 20th of September to the 20th of October* 1799; *being the result of the public and private Practice of a Physician at the West End of the Town.*

| | No. of Cases. | | No. of Cases. |
|---|---|---|---|
| ACUTE DISEASES. | | Hydrocephalus - - - | 2 |
| Contagious malignant Fever | 13 | Dyspepsia - - - - | 16 |
| Measles - - - - - | 5 | Gastrodynia - - - - | 10 |
| Small-pox - - - - | 2 | Entrodynia - - - - | 6 |
| Scarlet Fever - - - | 1 | Diarrhœa and Bilious Vomiting - - - | 14 |
| Hooping Cough - - | 6 | Menorrhagia - - | 4 |
| Catarrh - - - - - | 13 | Chlorosis and Amenorrhœa | 7 |
| Acute Rheumatism - | 2 | Ischuria - - - - | 1 |
| Ophthalmia - - | 3 | Enuresis - - - - - | 1 |
| Epistaxis - - - | 1 | Fluor Albus - - | 3 |
| Hæmoptoe - - - - | 1 | Cancer - - - - - - | 1 |
| Intestinal Hemorrhagy - | 1 | Tabes Mesenterica - - | 2 |
| Slow Fever - - | 1 | Worms - - - - | 2 |
| Hectic - - - - - | 3 | Hernia - - - - - | 2 |
| Childbed and Milk Fevers | 5 | Prolapsus Uteri - - | 1 |
| Acute Diseases of Infants | 11 | Scirrhus of the Liver - - | 1 |
| Erysipelas - - - - | 1 | Jaundice - - - - | 1 |
| Enteritis - - - - | 1 | Stricture of the Æsophagus | 1 |
| Cholera - - - - | 1 | Itch - - - - - | 6 |
| CHRONIC DISEASES. | | Prurigo - - - - | 2 |
| Cough and Dispnœa - | 20 | Nettle Rash - - - - | 2 |
| Phthisis pulmonalis - | 8 | Shingles - - - - | 1 |
| Chronic Rheumatism - | 5 | Impetigo - - - - - | 1 |
| Asthenia - - - - | 24 | Acne - - - - | 1 |
| Dropsy - - - - | 6 | Lupus - - - - - - | 1 |
| Scrophula - - - - | 3 | Erythema - - - - | 2 |
| Cephalœa - - - - | 8 | Porrigo - - - - - - | 5 |
| Hysteria - - - - - | 2 | Thrush - - - - | 4 |
| Epilepsy . - - - | 1 | | |

